# Supplementary material for: The domino effect of climate tipping points: a multidisciplinary perspective on global risks
Source: Natl Sci Rev. 2026 Jan 22;13(20):nwag042. doi: 10.1093/nsr/nwag042 (PMC13729756; doi:10.1093/nsr/nwag042)
Supplement: Supplementary file 1 [file nwag042_supplemental_file.pdf]

PERSPECTIVE

National Science Review
XX: output, Year
doi: https://doi.org/10.1093/nsr/XXXX

EARTH SCIENCES

The domino effect of climate tipping points: a multidisciplinary perspective on global risks

Jun Meng[1] and Deliang Chen[2,*]

Climate change is pushing Earth's interconnected systems toward critical thresholds. Several planetary boundaries such as those associated with climate regulation and biosphere integrity, have already been transgressed [1]. These violations heighten the risk of crossing climate tipping points, where small perturbations may induce abrupt and potentially irreversible shifts in major Earth subsystems. Tipping thresholds exist within the cryosphere, biosphere, and ocean circulation systems, each capable of exhibiting bi-stability and hysteresis, and reinforcing feedbacks under sustained anthropogenic forcing. Crucially, these tipping points rarely operate in isolation; instead, they may initiate cascading transitions across interconnected domains, amplifying systemic risks to ecosystems, economies, and societies. This perspective explores the cascading dynamics of climate tipping points, with a focus on marine ecosystems, and proposes a multidisciplinary framework using percolation theory and multilayer networks to better anticipate and manage these risks within a planetary boundaries framework.

The concept of climate tipping points emerged from paleoclimate studies in the 1980s, which revealed abrupt events like Dansgaard–Oeschger and Heinrich shifts. These suggested that small perturbations can trigger rapid, nonlinear transitions in Earth systems [2]. Building on this, Lenton et al. (2008) identified nine key tipping elements, such as Arctic summer sea-ice loss, Atlantic Meridional Overturning Circulation (AMOC) collapse, and Amazon rainforest dieback, with profound scientific and policy implications [3]. More recent research shows that these tipping elements are interlinked, forming a network in which the failure of one can cascade to others through physical and biogeochemical feedbacks [4]. For instance,

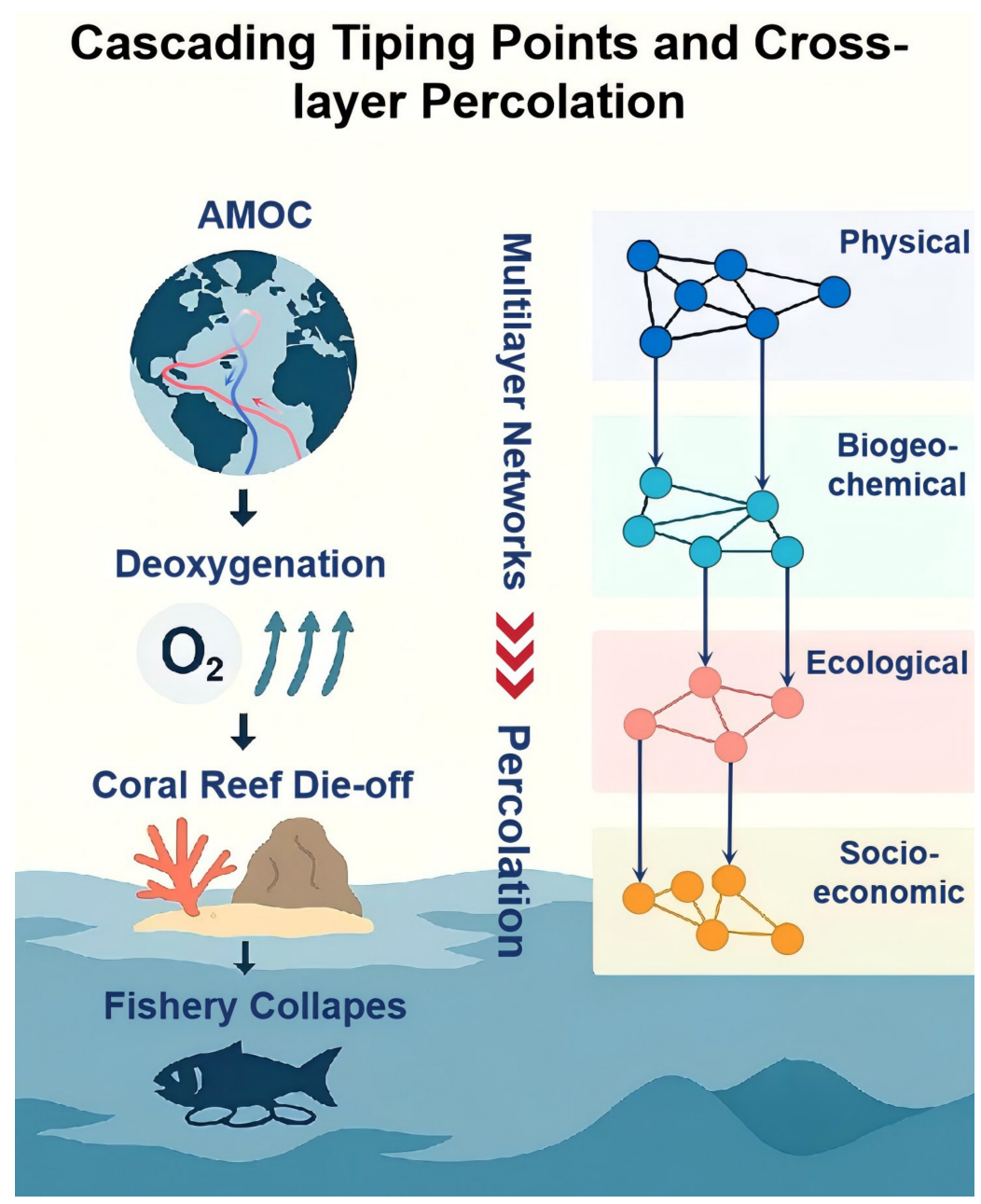

Figure 1. Cascading tipping points in a multilayer ocean system. This conceptual diagram illustrates how a tipping event in the physical ocean system—such as AMOC weakening—can trigger deoxygenation in the biogeochemical layer. This, in turn, may lead to coral reef die-off in the ecological layer and eventual fishery decline in the socioeconomic layer. These cross-domain, cross-scale transitions are driven by percolation-like processes through a multilayer network, where perturbations propagate between interconnected subsystems. The figure underscores the need to view ocean tipping elements as interdependent components of a complex, stratified Earth system, in which cascading failures can compromise climate regulation, biodiversity, and human well-being.

[1]State Key Laboratory of Earth System Numerical Modeling and Application, Institute of Atmospheric Physics, Chinese Academy of Sciences, Beijing, 100029, China; [2]Department of Earth System Science, Tsinghua University, Beijing 100084, China.;
[3]The research for this article was financed by the National Key R&D Program of China (2025YFF0517203), the National Natural Science Foundation of China (NSFC) (Grant Nos. 42575057, 12205025), and Tsinghua University (100008001).

*Corresponding authors. Email: deliangchen@tsinghua.edu.cn.

Received: XX XX Year; **Revised:** XX XX Year; **Accepted:** XX XX Year

© The Author(s) Year. Published by Oxford University Press on behalf of China Science Publishing & Media Ltd. This is an Open Access article distributed under the terms of the Creative Commons Attribution License (http://creativecommons.org/licenses/by/4.0/), which permits unrestricted reuse, distribution, and reproduction in any medium, provided the original work is properly cited.

Arctic sea-ice loss can weaken the AMOC, leading to Amazon dieback and further carbon release, potentially destabilizing the West Antarctic Ice Sheet. Such compound risks, highlighted in the Intergovernmental Panel on Climate Change Sixth Assessment Report (IPCC AR6), underscore the urgent need for integrated approaches to anticipate and address cascading tipping dynamics [5].

From a theoretical standpoint, climate tipping points are rooted in the principles of nonlinear dynamic systems and statistical physics, particularly bifurcation theory, phase transitions, and critical phenomena. Dynamic systems theory conceptualizes the climate as a multi-stable system, shaped by slow external forcing and internal feedbacks. As the system approaches a bifurcation point, such as a saddle-node or Hopf bifurcation, its resilience declines, increasing susceptibility to abrupt transitions between stable regimes [6]. Statistical physics complements this view by interpreting these transitions as phase changes driven by collective interactions. Systems near criticality exhibit heightened sensitivity to perturbations and fluctuations. Concepts such as correlation length, order parameters, and susceptibility—borrowed from condensed matter physics—can be applied to climate tipping elements to uncover universal properties of transition dynamics [7].

Tipping elements often form networks of coupled, multi-stable subsystems. Complex network theory helps reveal hidden interactions and long-range teleconnections in high-dimensional data, complementing dynamical models [8]. Within this context, percolation theory, rooted in statistical physics, offers a powerful tool to model how tipping events propagate. Once interdependencies exceed a critical threshold, local perturbations can trigger global cascades, akin to a fire sweeping through a dense forest [9]. This provides a conceptual foundation for analyzing systemic tipping risks that propagate across spatial and functional scales.

Beyond pairwise perspectives, it is increasingly important to adopt a broader framework that captures tipping dynamics across domains. In this regard, multilayer climate networks [9] offer a framework to quantify interactions both within and across Earth subsystems. For example, a multilayer network could link AMOC weakening (physical layer) to oxygen depletion (biogeochemical layer), coral reef collapse (ecological layer), and fishery losses (socioeconomic layer), revealing hidden teleconnections and compound vulnerabilities (Fig. 1).

Cascading interactions across scales and domains are particularly pronounced in the ocean system, which plays a central role in regulating planetary boundaries. Far from uniform, the ocean comprises a multilayer network of interdependent subsystems, including physical, biogeochemical, and ecological layers, each capable of undergoing distinct tipping dynamics [10]. Crucially, a tipping event in one layer can alter the boundary conditions of another, triggering cascading transitions across compartments.

Key ocean tipping elements—such as AMOC weakening, deoxygenation, coral reef collapse, and breakdown of the biological carbon pump—often arise not from abrupt shocks but from the slow, spatially heterogeneous accumulation of stressors like warming, acidification, and oxygen depletion. Though these drivers accumulate slowly and unevenly, their impacts may be sudden, nonlinear, and irreversible. The resulting cascades could undermine oceanic climate feedbacks, damage marine ecosystems, and endanger livelihoods, pushing the Earth system beyond safe operating limits. For example, collapse of carbon pump could raise atmospheric CO_2, while widespread fishery loss could threaten food security. Managing such compound risks calls for integrated approaches that bridge climate science, complex systems theory, and socioeconomic policy.

Anticipating tipping events remains a major scientific and policy challenge. Systems nearing critical transitions often display early-warning signals, such as critical slowing down, increased variance, and rising autocorrelation, that may precede abrupt shifts [11]. Yet the propagation of these risks across interconnected subsystems remains poorly understood. Current models struggle to capture nonlinear dynamics and emergent behavior, and observational systems often lack the necessary spatiotemporal resolution, especially in deep oceans or geopolitically sensitive regions.

Meeting these challenges requires a multidisciplinary agenda. Priorities include developing cross-compartment models that incorporate nonlinear dynamics and phase transition theory, constructing multilayer networks of coupled systems, and deploying real-time monitoring platforms. Equally important are robust early-warning detection frameworks and decision-making tools under uncertainty, informed by resilience theory, risk governance, and complex systems science, and supported by artificial intelligence (AI) to address the high complexity, nonlinearity, and multiscale nature of Earth sys-

tem dynamics.

Recent studies have begun to make progress along these lines. Quantitative approaches such as climate network analysis identify teleconnections and interaction pathways among potential tipping elements [8], while nonlinear dynamical models of interacting tipping points illustrate how coupling reshapes stability landscapes and induces cascading transitions [12,13]. More general theories of interdependent networks further suggest that cascading failures can exhibit characteristic precursor features, including transient plateau-like regimes and abrupt changes in connectivity [9,14]. In parallel, physics-based early-warning indicators derived from comprehensive climate models [15] and emerging AI-assisted diagnostics [16] demonstrate how mechanistic understanding and data-driven methods can be combined to assess proximity to critical transitions.

Despite these advances, most existing approaches remain largely diagnostic, with signals reflecting statistical dependencies or idealized dynamics under simplifying assumptions rather than precise prediction of real-world tipping events in specific Earth system components.

More fundamentally, advancing this agenda will require moving beyond the simple or parallel application of existing methods toward interdisciplinary approaches in which theoretical frameworks and technical tools are developed in tandem. Artificial intelligence illustrates this challenge: while existing AI techniques are increasingly influential, their full potential in tipping-point research will only be realized through theory-guided, complex-systems-aware frameworks, potentially inspired by principles from nonlinear dynamics, network science, and neuroscience. Realizing such integrative approaches will also require institutional and funding structures that better support genuinely transdisciplinary research, which remains difficult under predominantly discipline-oriented evaluation and funding systems. Addressing these structural constraints is therefore an essential complement to methodological innovation. Such efforts are critical for safeguarding marine ecosystems, protecting coastal communities, and maintaining the broader stability of Earth's planetary boundaries in a warming world.

REFERENCES

1. Rockström J, Steffen W, Noone K *et al.* A safe operating space for humanity. *nature* 2009; **461**: 472–475.
2. Steffen W, Jäger J, Carson DJ *et al. Challenges of a changing earth: proceedings of the global change open science conference, Amsterdam, The Netherlands, 10–13 July 2001*, (Springer Science & Business Media2002).
3. Lenton TM, Held H, Kriegler E *et al.* Tipping elements in the earth's climate system. *Proceedings of the national Academy of Sciences* 2008; **105**: 1786–1793.
4. Wunderling N, von der Heydt AS, Aksenov Y *et al.* Climate tipping point interactions and cascades: A review. *Earth System Dynamics* 2024; **15**: 41–74.
5. Masson-Delmotte V, Zhai P, Pirani A *et al.* Climate change 2021: the physical science basis. *Contribution of working group I to the sixth assessment report of the intergovernmental panel on climate change* 2021; **2**: 2391.
6. Yi C, Rietkerk M, Anderies JM *et al.* Principles for guiding future research on resilience and tipping points. *Environmental Research Letters* 2025; **20**: 031008.
7. Stanley HE. *Introduction to Phase Transitions and Critical Phenomena*, (Oxford University Press1987). Google-Books-ID: C3BzcUxoaNkC.
8. Liu T, Chen D, Yang L *et al.* Teleconnections among tipping elements in the earth system. *Nature Climate Change* 2023; **13**: 67–74.
9. Gross B, Volotsenko I, Sallem Y *et al.* The random cascading origin of abrupt transitions in interdependent systems. *Nature Communications* 2025; **16**: 5869.
10. Heinze C, Blenckner T, Martins H *et al.* The quiet crossing of ocean tipping points. *Proceedings of the National Academy of Sciences* 2021; **118**: e2008478118.
11. Scheffer M, Bascompte J, Brock WA *et al.* Early-warning signals for critical transitions. *Nature* 2009; **461**: 53–59.
12. Klose AK, Karle V, Winkelmann R *et al.* Emergence of cascading dynamics in interacting tipping elements of ecology and climate. *Royal Society open science* 2020; **7**: 200599.
13. Dekker MM, Von Der Heydt AS and Dijkstra HA. Cascading transitions in the climate system. *Earth System Dynamics* 2018; **9**: 1243–1260.
14. Buldyrev SV, Parshani R, Paul G *et al.* Catastrophic cascade of failures in interdependent networks. *Nature* 2010; **464**: 1025–1028.
15. Van Westen RM, Kliphuis M and Dijkstra HA. Physics-based early warning signal shows that amoc is on tipping course. *Science advances* 2024; **10**: eadk1189.
16. Bury TM, Sujith R, Pavithran I *et al.* Deep learning for early warning signals of tipping points. *Proceedings of the National Academy of Sciences* 2021; **118**: e2106140118.
